# Perioperative substance P/NK1R pharmacology: from PONV control to a testable cancer neuro-immune hypothesis

**DOI:** 10.3389/fimmu.2026.1896684

**Published:** 2026-07-16

**Authors:** Jing Zhao, Zhongwei Liu, Jun Wang

**Affiliations:** 1 Department of Anesthesiology, Shaanxi Provincial People’s Hospital, Xi’an, Shaanxi, China; 2 Department of Cardiology, Shaanxi Provincial People’s Hospital, Xi’an, Shaanxi, China; 3 Department of Anesthesiology, Shaanxi Provincial Cancer Hospital, Xi’an, Shaanxi, China

**Keywords:** cancer metastasis, immune checkpoint resistance, innate immunity, neurokinin-1 receptor, perioperative care, postoperative nausea and vomiting, substance P, tumor microenvironment

## Abstract

The perioperative period is increasingly recognized as a biologically active interval in which surgical injury, anesthetic exposure, neuroendocrine stress, pain, opioid use, and innate immune remodeling may influence residual tumor-cell ecology. Postoperative nausea and vomiting (PONV) provides a clinically validated perioperative phenotype of substance P (SP)/neurokinin-1 receptor (NK1R; TACR1) pharmacology, because NK1R antagonists are established antiemetic agents for selected high-risk patients. The relevance of this pharmacology to cancer does not rest on a shared PONV-tumor mechanism; rather, it rests on the fact that the same druggable receptor can be targeted within familiar perioperative dosing windows. PONV is therefore used here as a clinical pharmacology entry point to consider whether perioperative SP/NK1R signaling may have additional relevance as a candidate neuro-innate immune circuit within the tumor microenvironment. Recent evidence supports biological plausibility, but the mechanistic core remains narrow. Sensory-neuron-derived SP can activate tumoral TACR1 and extracellular RNA/TLR7-dependent metastatic programs in breast cancer models. A separate cancer-induced nerve-injury model supports the broader concept that neural injury can affect checkpoint responsiveness, but its IL-6/type 1 interferon/ATF-3 mechanism is distinct from SP/NK1R signaling. These findings fit a broader cancer-neuroscience framework in which tumor-associated nerves, neuropeptides, macrophages, neutrophil extracellular traps, extracellular RNA sensing, and inflammatory myeloid states may interact during perioperative stress. These contextual observations should not be read as direct SP/NK1R-specific evidence. Nevertheless, antiemetic efficacy should not be equated with proven anticancer efficacy. Available evidence remains dominated by a single causal breast cancer model, tumor expression studies, and emerging clinical associations; prospective perioperative trials have not established that NK1R blockade reduces recurrence, metastasis, or immunotherapy resistance. The next step should be biomarker-embedded perioperative research integrating PONV phenotyping, circulating SP, TACR1 expression, neutrophil extracellular trap markers, monocyte activation, extracellular RNA/TLR signatures, spatial tumor innervation, minimal residual disease readouts, and long-term oncologic outcomes. SP/NK1R signaling should therefore be viewed as a plausible and clinically tractable perioperative translational hypothesis, not yet as a validated oncologic intervention.

## 1 Introduction

The perioperative period is not a passive interval between diagnosis and adjuvant therapy. It is a concentrated neuroendocrine, inflammatory, metabolic, and hemostatic stress state that occurs precisely when residual tumor cells, micrometastatic niches, and immune surveillance are being reshaped. PONV is among the most visible patient-centered complications of this period and has generated a mature clinical pharmacology literature in which NK1R antagonists occupy a defined, evidence-based role (1).

The oncologic relevance of the same interval is less settled but biologically compelling. Surgery remains a mainstay for solid tumors, and perioperative perturbations may alter minimal residual disease through immune, neuroendocrine, inflammatory, and coagulation pathways (2). This does not mean that every perioperative drug modifies cancer outcomes. It does mean that perioperative interventions with immunological or neurobiological effects deserve mechanism-directed evaluation rather than being considered only as symptomatic therapies.

The connection between PONV and cancer in this Review is pharmacological rather than mechanistic. In anesthesia, NK1R antagonists are studied because SP is a key emetic neurotransmitter. In cancer biology, a recent Nature study identified neuronal SP as a driver of breast tumor growth, invasion, and metastasis through a TACR1-extracellular RNA-TLR7 axis, with the approved anti-nausea drug aprepitant suppressing tumor growth and metastasis in preclinical models (3). This discovery does not establish perioperative anticancer benefit, but it makes the SP/NK1R axis a rational candidate for translational perioperative investigation because the receptor is druggable within clinically familiar perioperative exposure windows.

The broader field of cancer neuroscience is also moving from descriptive tumor innervation toward mechanistic neuroimmune hypotheses. Cancer-induced nerve injury has been associated with resistance to anti-PD-1 therapy in cutaneous squamous-cell carcinoma, melanoma, and gastric cancer (4). Importantly, that study identified an IL-6- and type 1 interferon-driven inflammatory mechanism involving ATF-3-dependent programs and reversal by IL-6 blockade; it should therefore be interpreted as conceptual evidence for neural-immune checkpoint biology, not as SP/NK1R-specific evidence. SP/NK1R signaling should therefore be examined not as an isolated antiemetic mechanism but as one candidate component of a neuro-innate immune interface that may link sensory neurons, injured tissues, innate immune cells, extracellular nucleic-acid sensing, and tumor adaptation.

**Figure 1:**
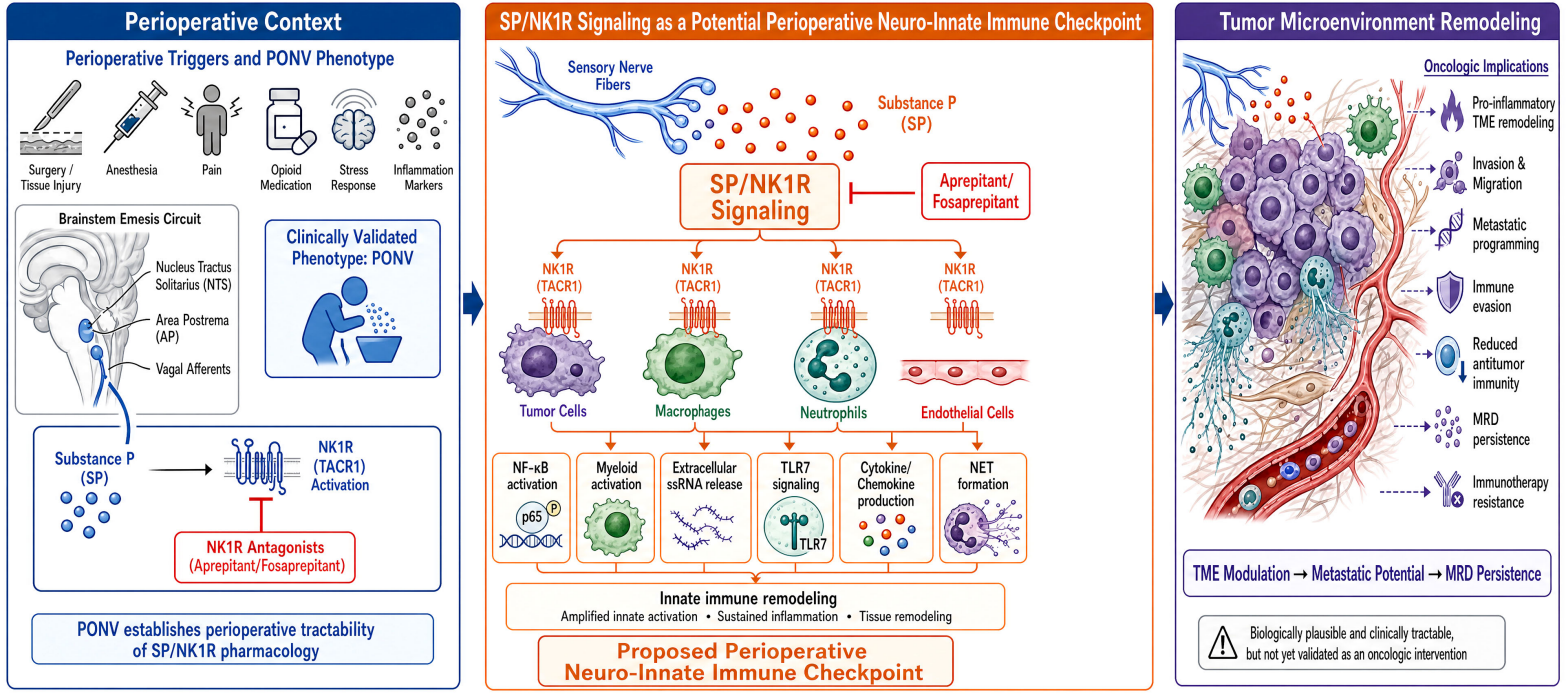
Perioperative SP/NK1R pharmacology as a clinical entry point for a testable cancer neuro-immune hypothesis. This schematic depicts a proposed framework in which perioperative triggers, including surgery, tissue injury, anesthesia, pain, opioid exposure, stress responses, and inflammation, converge on SP/NK1R-dependent biology. PONV represents a clinically validated phenotype of this pathway through SP-mediated NK1R activation within brainstem emetic circuitry and its blockade by aprepitant or fosaprepitant. This shared pharmacology does not establish a shared mechanism between emesis and cancer biology. Beyond emesis, sensory nerve fibers and tumor-associated neural inputs may release SP in the perioperative tumor milieu. SP/NK1R signaling may influence tumor cells, macrophages, neutrophils, and endothelial cells, thereby promoting inflammatory signaling, myeloid activation, extracellular ssRNA release, TLR7 signaling, cytokine and chemokine production, and NET formation. These processes provide a plausible route toward innate immune remodeling, tumor microenvironment modulation, metastatic programming, minimal residual disease persistence, and potential immunotherapy resistance. The oncologic relevance of this framework remains to be prospectively validated. AP, area postrema; MRD, minimal residual disease; NET, neutrophil extracellular trap; NK1R, neurokinin-1 receptor; NTS, nucleus tractus solitarius; PONV, postoperative nausea and vomiting; SP, substance P; ssRNA, single-stranded RNA; TACR1, tachykinin receptor 1; TLR7, Toll-like receptor 7; TME, tumor microenvironment.

This Review critically evaluates SP/NK1R signaling as a provisional perioperative neuro-innate immune checkpoint in cancer. Here, “neuro-innate immune checkpoint” is used as a working term, not established nomenclature, to describe a clinically targetable neural-innate immune interface whose modulation could be tested using perioperative pharmacodynamic biomarkers. The term does not imply equivalence with T-cell immune checkpoints or proven anticancer efficacy. It uses PONV as the clinical entry point, then integrates pharmacological, immunological, and cancer-neuroscience evidence to propose a testable translational framework. The central argument is deliberately cautious: NK1R antagonism is clinically validated for prevention of PONV, biologically plausible as a tumor-microenvironment modulator, and insufficiently proven as an oncologic intervention. The central conceptual framework is shown in **Figure 1**. The review is organized to move from the perioperative stress context and validated PONV pharmacology to SP/NK1R immune biology, tumor-neural mechanisms, clinical controversies, and a staged biomarker-driven translational roadmap.

## 2 The perioperative interval as a neuroimmune stress test in cancer

For cancer surgery, the perioperative interval should be considered a short but high-density biological perturbation. Tissue injury, pain, anesthesia, opioids, catecholamines, glucocorticoids, platelet activation, neutrophil mobilization, and acute-phase inflammation converge during a period in which occult residual tumor cells may be exposed to altered immune pressure. The major conceptual advance is not that surgery inevitably causes recurrence, but that perioperative biology may influence which residual tumor cells survive and which immune programs dominate early after resection (2).

Clinically, this concept remains difficult to prove because recurrence is delayed, heterogeneous, and strongly shaped by tumor stage, biology, and adjuvant therapy. This limitation should prevent overinterpretation of perioperative interventions. Nevertheless, mechanism-based perioperative studies are valuable because they can measure early pharmacodynamic endpoints that occur before survival endpoints become interpretable. SP/NK1R signaling is well suited to this approach because perioperative NK1R antagonists already exist, dosing windows are clinically familiar, and PONV provides an immediate pharmacodynamic phenotype.

## 3 PONV: a clinically validated phenotype of SP/NK1R pharmacology

PONV is multifactorial, but it is one of the most reproducible clinical settings in which the SP/NK1R pathway has been pharmacologically tested. The Apfel simplified risk score established four readily measurable predictors: female sex, non-smoking status, history of PONV or motion sickness, and postoperative opioid use (5). For this Review, the value of the Apfel score is not that it identifies SP/NK1R pathway activation; rather, it shows that PONV risk reflects patient susceptibility and perioperative exposure. This distinction prevents PONV risk from being misread as a receptor-specific biomarker while preserving PONV as a clinically useful setting for studying NK1R antagonist pharmacology.

The first large randomized, double-blind trial comparing oral aprepitant with intravenous ondansetron enrolled 805 patients receiving general anesthesia for open abdominal surgery. Aprepitant did not improve the composite complete-response endpoint, but it was superior to ondansetron for preventing vomiting during the first 24 and 48 postoperative hours, while nausea control and rescue antiemetic use were not significantly different (6). This separation between vomiting and nausea is mechanistically important. It indicates that NK1R blockade is particularly strong against emetic output but does not fully suppress the subjective and multifactorial experience of nausea.

A second randomized, double-blind phase III trial in major abdominal surgery similarly found that aprepitant was non-inferior to ondansetron for complete response and superior for no vomiting at 24 and 48 hours (7). These trials support NK1R antagonism as an anti-vomiting strategy, but they also constrain translational claims. If the clinical phenotype most directly linked to NK1R blockade is vomiting rather than all nausea, then perioperative oncology studies should avoid using PONV improvement alone as evidence of broader immune or tumor modulation.

More recent evidence has tested intravenous fosaprepitant in combination prophylaxis. In a randomized trial of 1,154 high-risk patients undergoing laparoscopic gastrointestinal surgery, fosaprepitant added to dexamethasone and palonosetron reduced PONV during the first 24 hours compared with placebo plus the same background regimen, but intraoperative hypotension was more frequent in the fosaprepitant group (8). This trial is especially relevant for cancer surgery because it demonstrates feasibility of NK1R antagonist integration into modern multimodal prophylaxis, while reminding investigators that perioperative drug repurposing must preserve anesthetic safety.

## 4 Clinical evidence supports antiemesis, not yet anticancer activity

A systematic review and meta-analysis of NK1R antagonists for PONV concluded that this class was effective for preventing postoperative vomiting and reducing rescue antiemetic use across randomized trials (9). This synthesis strengthens the antiemetic evidence base but does not address recurrence, metastasis, or immune remodeling.

A separate systematic review and meta-analysis focused on aprepitant found evidence supporting its use for PONV prevention and highlighted its particular efficacy against vomiting (10). For perioperative oncology, the implication is practical: if aprepitant or fosaprepitant is studied as a tumor-microenvironment intervention, investigators can ethically embed it within accepted antiemetic practice while measuring tumor-relevant endpoints independently of symptom control.

Regulatory labeling also imposes boundaries on interpretation. DailyMed/FDA labeling states that aprepitant capsules are indicated in adults for prevention of PONV, that aprepitant has not been studied for treatment of established nausea and vomiting, and that chronic continuous administration is not recommended because it has not been studied (11). These restrictions matter for oncology translation because perioperative window dosing is more defensible than prolonged off-label exposure without pharmacokinetic, pharmacodynamic, and safety justification. The evidence layers that support, limit, and contextualize this interpretation are summarized in **Table 1**.

**Table 1:** Evidence layers supporting SP/NK1R as a perioperative translational hypothesis.

Evidence layer	What is supported	What is not yet supported	Implication for review interpretation
PONV randomized trials and guidelines	NK1R antagonism prevents postoperative vomiting and can be used within multimodal prophylaxis in selected patients.	Anticancer effects, recurrence reduction, or immune reprogramming.	Use PONV as a clinical pharmacology anchor, not as a tumor surrogate.
Structural and signaling biology	NK1R is a druggable GPCR with characterized antagonist binding and context-dependent signaling.	Prediction of tumor response from receptor expression alone.	Mechanistic studies need cell-specific pharmacodynamics.
Immune-cell studies	SP/NK1R can modulate monocyte/macrophage programs, cytokines, and tissue-factor biology.	Clinical suppression of perioperative inflammation by approved antiemetic dosing.	Measure innate immune biomarkers in perioperative studies.
Tumor-neuroscience models	Neuronal SP can promote tumor growth and metastasis through TACR1-dependent pathways in selected models.	Generalizable benefit across all cancers or all perioperative settings.	Prioritize tumor types with measurable candidate neuroimmune pathway features, while validating assays prospectively.
Clinical neuroimmune oncology	Tumor-associated nerve injury can correlate with poor immunotherapy response in some cancers.	SP/NK1R as the dominant mediator of immune-checkpoint resistance.	Test SP/NK1R within a broader neural-immune network.

Oral aprepitant is most suitable when preoperative administration and postoperative oral absorption are feasible, whereas intravenous fosaprepitant is practical when oral dosing is undesirable or when single perioperative administration is preferred (8). These formulation differences should be interpreted as clinical and pharmacologic considerations, not as evidence that either agent has tumor-specific activity (11).

## 5 SP/NK1R signaling as a neuro-innate immune module

SP is a tachykinin neuropeptide with major roles in nociception, vascular responses, inflammation, and emesis. NK1R is its high-affinity receptor and belongs to the G protein-coupled receptor family; tachykinin biology is characterized by ligand-receptor promiscuity, receptor internalization, and context-dependent signaling rather than by a single linear pathway (12). This complexity is an advantage for physiology but a challenge for therapeutic interpretation.

Contemporary structural and signaling studies have refined this view. Cryo-electron microscopy and signaling analyses showed that SP-bound NK1R can engage multiple G proteins, and that related peptides can bias signaling through the same receptor (13). This means that NK1R antagonism in the perioperative setting may block only part of a broader tachykinin network, and that receptor expression alone is unlikely to predict biological effect without information about ligand source, local concentration, receptor isoform, cellular context, and downstream pathway engagement.

**Figure 2:**
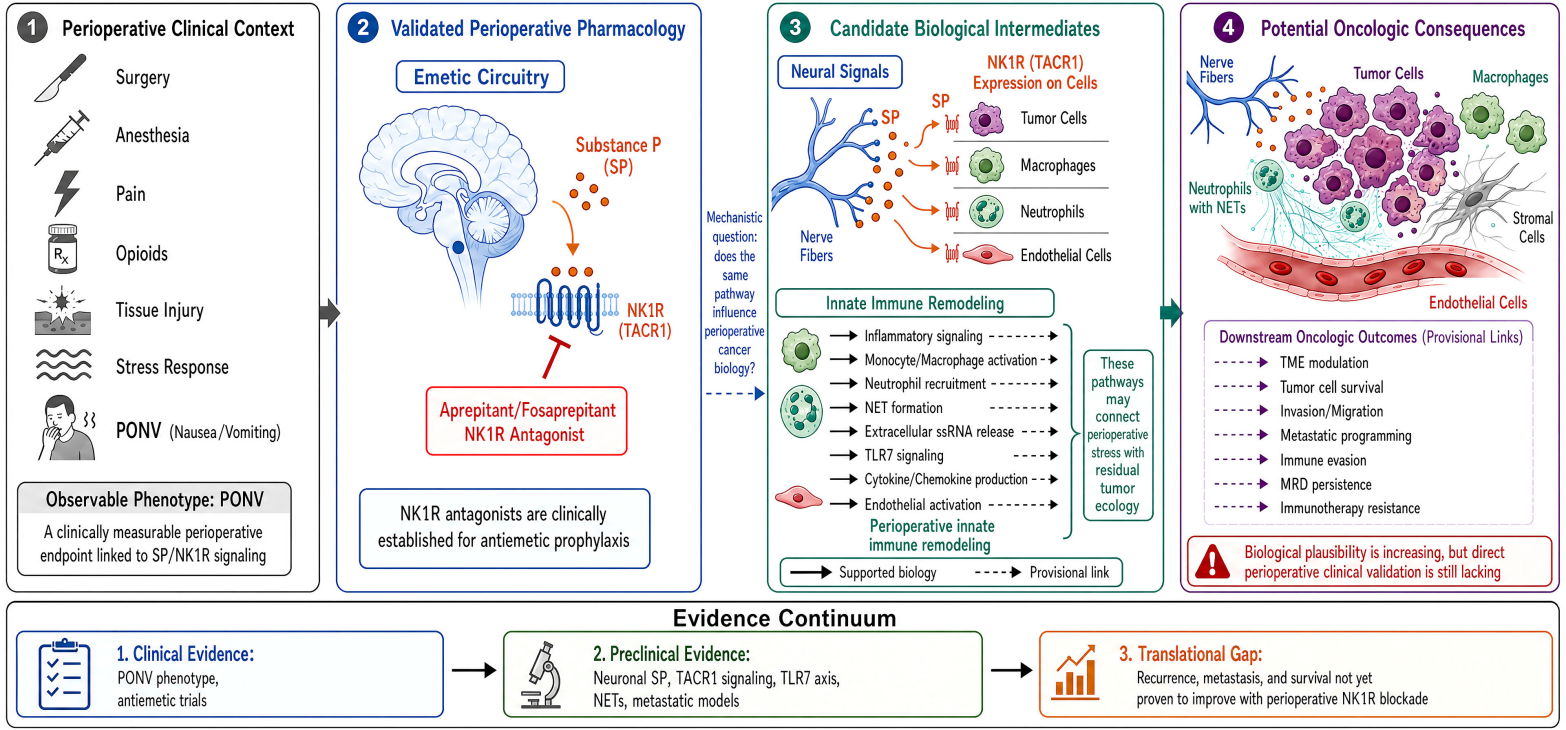
Translational bridge from PONV pharmacology to tumor microenvironment modulation. This schematic connects perioperative clinical observation with candidate mechanisms of tumor microenvironment remodeling. The left modules summarize perioperative triggers and the clinically measurable PONV phenotype, followed by validated SP/NK1R antiemetic pharmacology in which aprepitant or fosaprepitant block NK1R signaling in emetic circuitry. The middle module extends this framework to biological intermediates, including sensory nerve-derived SP, TACR1 expression on tumor and innate immune cells, inflammatory signaling, monocyte/macrophage activation, neutrophil recruitment, NET formation, extracellular ssRNA release, TLR7 signaling, cytokine and chemokine production, and endothelial activation. The right module summarizes provisional oncologic consequences, including tumor cell survival, invasion and migration, metastatic programming, immune evasion, MRD persistence, and possible immunotherapy resistance. The lower evidence continuum distinguishes established clinical evidence for PONV pharmacology from preclinical and contextual neuroimmune evidence, and it marks the perioperative-oncology link as unresolved. Solid arrows indicate supported biology, and dashed arrows indicate provisional perioperative-oncology links. MRD, minimal residual disease; NET, neutrophil extracellular trap; NK1R, neurokinin-1 receptor; PONV, postoperative nausea and vomiting; SP, substance P; ssRNA, single-stranded RNA; TACR1, tachykinin receptor 1; TLR7, Toll-like receptor 7; TME, tumor microenvironment.

Crystal structures of human NK1R bound to aprepitant, netupitant, and CP-99,994 provide a molecular basis for antagonist recognition and receptor conformational control (14). These data are relevant to drug repurposing because they show that clinically used antiemetics are not vague neuromodulators; they are structurally characterized receptor antagonists. However, structural target engagement does not define which tumor or immune compartment is dominant *in vivo*. The translational bridge from PONV pharmacology to tumor microenvironment modulation is depicted in **Figure 2**.

Immunologically, SP is more than a neuronal pain mediator. Reviews of SP immunobiology describe effects on immune-cell trafficking, cytokine release, mast-cell activation, dendritic cells, T cells, macrophages, and barrier inflammation (15). These observations justify considering SP/NK1R as a neuroimmune axis, but they also reveal why simple pro-inflammatory or anti-inflammatory labels are inadequate.

Human monocytes and macrophages can express both SP and NK1R transcripts, supporting the possibility of autocrine or paracrine tachykinin signaling within innate immune compartments (16). This finding is important for perioperative cancer because monocytes and macrophages respond rapidly to tissue injury, interact with coagulation and complement systems, and can influence tumor-cell dissemination and metastatic niche formation.

In a human monocyte study, SP-NK1R interaction upregulated tissue factor and induced cytokine and chemokine release, while NK1R inhibition blocked tissue-factor expression (17). This experiment connects SP/NK1R signaling to coagulation-linked innate immune activation. In perioperative oncology, where thrombosis, inflammation, and metastasis intersect, such data support a mechanistic hypothesis rather than a clinical conclusion.

## 6 Innate immune effector circuits: neutrophils, extracellular traps, and postoperative metastasis

NET studies are included here as perioperative innate-immunity context rather than as direct evidence that SP/NK1R signaling controls NET biology. Neutrophils are a central perioperative innate immune population because they respond rapidly to tissue injury and can form neutrophil extracellular traps (NETs). In a liver-metastasis model, surgical stress promoted NET formation, and NETs facilitated metastatic development and progression (18). This finding does not implicate SP/NK1R directly, but it identifies a perioperative innate immune effector system that could plausibly intersect with neuropeptide signaling.

Clinical relevance is suggested by gastric cancer data linking postoperative abdominal infectious complications, NET formation, and metastatic progression (19). These observations again do not prove that NK1R blockade modifies NET biology in patients. They do indicate that perioperative inflammation and innate immune activation can have tumor-relevant consequences, which is the biological context in which SP/NK1R should be tested.

## 7 Tumor innervation and the SP/TACR1/extracellular RNA-TLR7 axis

The tumor-specific evidence is heterogeneous, and the mechanistic core of the current SP/NK1R oncology argument rests most strongly on one breast cancer model. Evidence from colorectal, lung, and pancreatic cancers should therefore be interpreted as supportive context, expression association, or distinct pathway biology rather than as proof of a uniform SP/NK1R mechanism.

The most direct cancer-mechanistic evidence for SP/NK1R signaling comes from breast cancer models. Padmanaban and colleagues found that highly metastatic mammary tumors acquired increased innervation, that breast cancer cells induced sensory-neuron calcium activity and SP release, and that neuronal SP promoted tumor growth, invasion, and metastasis through tumoral TACR1 (3). This study is pivotal because it links a clinically targetable antiemetic receptor to a defined tumor-neuron interaction.

The same work identified an unexpected downstream mechanism: SP signaling induced death of a TACR1-high cancer-cell subset, extracellular single-stranded RNAs released from dying cells activated tumoral TLR7, and the resulting gene-expression program promoted metastatic behavior (3). The key translational insight is not simply that aprepitant inhibited tumor growth in mice. It is that SP/NK1R signaling may couple neuronal activity to innate nucleic-acid sensing within cancer cells.

Expression studies in human cancers provide supportive but lower-level evidence. In colorectal cancer tissue microarrays, SP and NK1R expression were upregulated compared with adjacent normal tissues and were associated with tumor progression and prognosis (20). Such observational data cannot establish causality, but they help identify tumor types in which TACR1 expression and SP-rich microenvironments may warrant mechanistic evaluation. In non-small-cell lung cancer, NK1R promoted tumor progression through transactivation of epidermal growth factor receptor signaling in experimental models (21). This study expands the pathway beyond breast cancer and suggests that SP/NK1R may cooperate with classical receptor-tyrosine-kinase programs. However, tumor-intrinsic growth signaling and perioperative neuroimmune signaling remain distinct questions and should not be merged without direct experimental evidence. Pancreatic cancer provides an additional neurotropic context. A study of SP/NK1R signaling in pancreatic cancer perineural invasion reported that the SP/NK1R axis promoted perineural invasion through a long non-coding RNA-mediated mechanism (22). This supports the relevance of neural-tumor interactions in pancreatic cancer, but it represents a mechanism different from the breast cancer extracellular RNA/TLR7 axis.

Countervailing findings also require attention. In hepatoblastoma, truncated TACR1 was broadly expressed but did not correlate with investigated tumor characteristics, including outcome (23). In pancreatic ductal adenocarcinoma datasets, one study reported lower NK1R expression in tumor tissue and a better overall-survival signal among patients with higher NK1R expression, despite growth-reduction effects of aprepitant in cell models (24). These findings show that TACR1/NK1R expression is not a uniform adverse biomarker and that pathway dependence cannot be inferred from receptor detection alone.

Across tumor types, the putative SP/NK1R mechanism appears heterogeneous. In breast cancer, the strongest evidence links sensory-neuron-derived SP to tumoral TACR1 and extracellular RNA/TLR7-dependent metastasis (3). In colorectal cancer, current evidence is mainly expression-prognosis association rather than causal pathway dependence (20). In lung cancer, available models suggest crosstalk between NK1R and epidermal growth factor receptor signaling (21). In pancreatic cancer, the axis is most closely linked to perineural invasion and neurotropic growth patterns (22). These differences should be treated as a central design constraint for perioperative studies rather than as a minor limitation.

## 8 From tumor-neuron crosstalk to immune-checkpoint resistance

The tumor immune microenvironment integrates cancer cells, fibroblasts, endothelial cells, neurons, myeloid cells, lymphocytes, extracellular matrix, metabolites, and therapy-induced stress. A framework for understanding the tumor immune microenvironment emphasizes that effective therapy depends on the spatial and functional organization of these cellular and molecular components, not only on the presence of T cells (25). SP/NK1R signaling fits this framework because it may affect neuronal input, innate sensing, and tumor-cell stress responses simultaneously.

Immune-checkpoint resistance is similarly multifactorial. Primary, adaptive, and acquired resistance to immunotherapy can arise from tumor-cell-intrinsic defects, inadequate antigen presentation, suppressive myeloid programs, stromal exclusion, interferon pathway rewiring, and host factors (26). SP/NK1R signaling should therefore be studied as one candidate modifier of the resistance ecosystem, not as a stand-alone explanation for immunotherapy failure.

Cancer-induced nerve injury strengthens the broader rationale for studying neural-immune interfaces, but it does not validate SP/NK1R blockade. Baruch and colleagues reported that perineural invasion and cancer-induced nerve injury were associated with poor response to anti-PD-1 therapy across cutaneous squamous-cell carcinoma, melanoma, and gastric cancer (4). Mechanistically, their study implicated IL-6 and type 1 interferon-driven inflammation, ATF-3-dependent programs, and reversibility by IL-6 blockade, which are distinct from the SP/TACR1/extracellular RNA/TLR7 pathway. The relevance of this work to the present framework is therefore conceptual: it supports biomarker-based testing of neural-immune circuits, not the assumption that SP/NK1R is the dominant mediator of checkpoint resistance.

Established immunotherapy-resistance reviews distinguish tumor-intrinsic and tumor-extrinsic mechanisms and highlight myeloid suppression, inflammatory cytokines, and stromal barriers as recurrent themes (27). SP/NK1R could intersect with these themes through monocyte/macrophage activation, endothelial effects, neurogenic inflammation, and extracellular RNA/TLR signaling. The evidence is sufficiently coherent to justify targeted research, but not sufficiently mature to support routine oncologic use of NK1R antagonists.

## 9 Contextual cancer-neuroscience and innate-immunity evidence: defining the hypothesis space

The following cancer-neuroscience and innate-immunity studies define the biological environment in which a perioperative SP/NK1R hypothesis could be tested. They should not be read as cumulative SP/NK1R-specific evidence, because many of these models involve adrenergic, cholinergic, growth-factor, cytokine, or neuroligin-mediated mechanisms.

The expanded cancer-neuroscience literature supports the view that neural signaling is a component of the tumor microenvironment rather than a distant host factor (28). A field-level roadmap has argued that bidirectional communication between cancers and the nervous system is sufficiently mature to justify coordinated mechanistic and translational research (29). Several tumor contexts illustrate the breadth of this principle without establishing SP/NK1R dependence. Loss of TP53 can drive adrenergic transdifferentiation of tumor-associated sensory nerves in head-and-neck cancer (30). Autonomic nerve development can regulate prostate cancer initiation and dissemination in mouse models (31), and adrenergic nerves can activate an endothelial angio-metabolic switch in prostate cancer (32). Aberrant cholinergic signaling has been implicated in gastric tumorigenesis (33), while neuronal activity can stimulate high-grade glioma growth through neuroligin-3 secretion (34). These examples justify measuring neural inputs in perioperative studies, but they also emphasize that SP/NK1R is only one candidate pathway within a broader neural network.

Pancreatic cancer is particularly relevant to a perioperative neuroimmune model because sensory nerves, pain biology, and perineural invasion are prominent. Sensory-neuron ablation delayed initiation and progression of pancreatic ductal adenocarcinoma in a genetic mouse model (35). Neuroplastic changes may occur early during pancreatic ductal adenocarcinoma development (36). Stress biology provides another systemic bridge: sympathetic nervous system activation induced a metastatic switch in primary breast cancer models through beta-adrenergic signaling and macrophage-related prometastatic gene expression (37). A broader synthesis concluded that autonomic signaling can regulate macrophage infiltration, inflammation, angiogenesis, epithelial-mesenchymal transition, invasion, cellular immunity, and programmed cell death within tumor microenvironments (38).

The innate-immunity component of the perioperative hypothesis is strengthened by NET-metastasis studies. NETs trapped circulating tumor cells and promoted metastasis in murine infection models, with effects reduced by DNase or neutrophil elastase inhibition (39). Inflammation-induced NETs can also awaken dormant disseminated cancer cells through protease-dependent extracellular-matrix remodeling and integrin-mediated signaling (40). These data justify NET markers as candidate perioperative endpoints, but they do not demonstrate that NK1R antagonists regulate NET formation in patients.

The immunotherapy-resistance implications should be framed with equal caution. Stromal architecture can exclude T cells from cancer-cell contact and limit cytotoxic responses (41). Pan-tumor biomarker analyses of PD-1 blockade have shown that tumor mutational burden and T-cell-inflamed gene-expression profiles capture complementary dimensions of response biology (42). A perioperative SP/NK1R trial should therefore integrate neural, innate immune, stromal, and T-cell-inflamed readouts rather than rely on a single neuropeptide marker. Collectively, these studies make the perioperative hypothesis testable, but they do not prove that perioperative NK1R antagonism improves cancer outcomes.

## 10 Clinical and translational implications

The first translational implication is pragmatic: perioperative NK1R antagonists can be studied without inventing a new drug class. Aprepitant is already approved for prevention of PONV in adults, and fosaprepitant can be incorporated into perioperative prophylaxis protocols in trial settings. This lowers the barrier to biomarker-rich window-of-opportunity studies, but it does not remove the need for safety monitoring, drug-interaction assessment, and tumor-specific rationale.

The second implication is biomarker selection. Candidate biomarkers should include circulating SP, TACR1 expression in tumor and immune compartments, tumor innervation density, pain and PONV phenotypes, monocyte tissue-factor expression, NET markers, cytokines, extracellular RNA signatures, TLR7 pathway activation, ctDNA dynamics, and minimal residual disease. These endpoints should be temporally sampled before surgery, immediately after surgery, at early postoperative follow-up, and during adjuvant therapy where feasible, as demonstrated in **Table 2**.

**Table 2:** Candidate perioperative biomarkers for SP/NK1R translational studies.

Biomarker domain	Candidate readouts	Preferred sampling window	Interpretive caution
Clinical phenotype	Vomiting episodes, nausea severity, rescue antiemetics, pain scores, opioid exposure.	Preoperative risk assessment; 0–24 h and 24–48 h after surgery.	Reduced vomiting is not evidence of tumor modulation.
Neuropeptide axis	Plasma SP, tumor SP immunostaining, TACR1 expression, prevalidated NK1R isoform-specific assays.	Baseline; immediate postoperative period; resected tumor tissue.	Bulk expression does not identify source-target pairs.
Innate immunity	Neutrophil count, NET markers, monocyte tissue factor, cytokine panels, C-reactive protein.	Baseline; 6–24 h; postoperative day 2-3.	Inflammation can reflect surgical trauma or complications rather than tumor biology.
Tumor spatial biology	Nerve density, sensory-neuron markers, TACR1-positive tumor cells, macrophage/neutrophil proximity, TLR7 signature.	Resected tumor and margin tissue.	Spatial heterogeneity requires standardized tissue sampling.
Minimal residual disease	ctDNA, circulating tumor cells, early molecular recurrence markers.	Baseline, early postoperative period, adjuvant-therapy milestones.	MRD dynamics are tumor-type and assay dependent.

**Figure 3:**
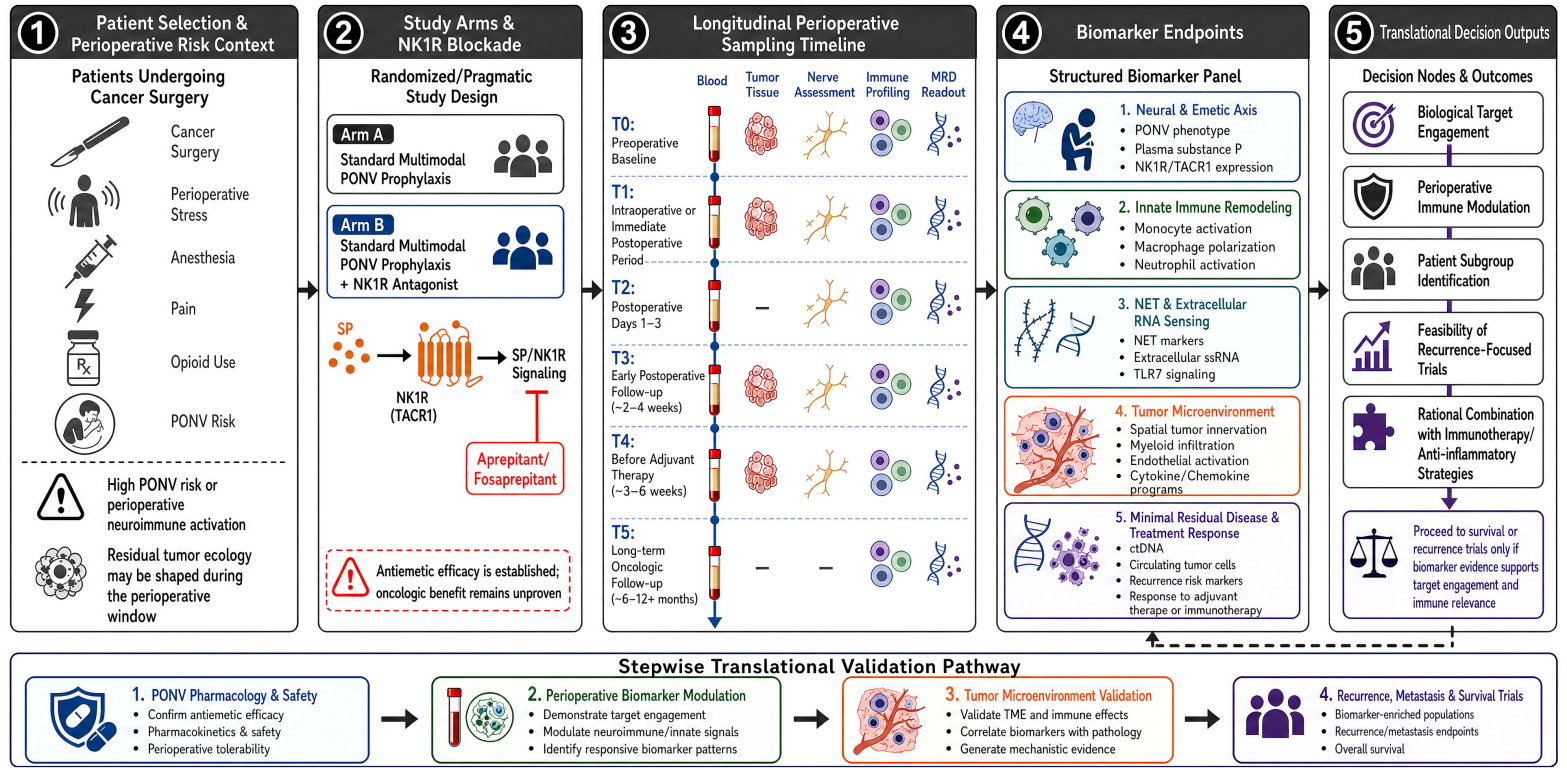
Biomarker-embedded perioperative trial framework for evaluating NK1R blockade in cancer. This schematic outlines a staged translational design for testing whether perioperative NK1R/TACR1 blockade has biological relevance beyond PONV prophylaxis. The framework begins with patients undergoing cancer surgery, in whom surgical injury, anesthesia, pain, opioid exposure, perioperative stress, and PONV risk may coincide with transient neuroimmune activation. A randomized or pragmatic design could compare standard multimodal PONV prophylaxis with standard prophylaxis plus an NK1R antagonist such as aprepitant or fosaprepitant. Longitudinal sampling across preoperative, immediate postoperative, postoperative day 1-3, early follow-up, pre-adjuvant therapy, and long-term follow-up windows would integrate blood, tumor tissue, peri-tumoral nerve assessment, immune profiling, and minimal residual disease readouts. Candidate endpoints include the neural and emetic axis, innate immune remodeling, NET and extracellular RNA sensing pathways, tumor microenvironment features, and minimal residual disease or treatment-response markers. Clinical development should proceed from PONV pharmacology and safety to perioperative biomarker modulation, tumor microenvironment validation, and only then to recurrence, metastasis, or survival trials. ctDNA, circulating tumor DNA; MRD, minimal residual disease; NET, neutrophil extracellular trap; NK1R, neurokinin-1 receptor; PONV, postoperative nausea and vomiting; SP, substance P; ssRNA, single-stranded RNA; TACR1, tachykinin receptor 1; TLR7, Toll-like receptor 7; TME, tumor microenvironment.

The third implication is trial design. A definitive recurrence endpoint is premature without pharmacodynamic evidence that perioperative NK1R blockade modifies relevant tumor or immune biology. A more appropriate first step is a randomized, biomarker-embedded perioperative study in a tumor type with evidence of innervation, TACR1 expression, or SP/TLR activity. Such a trial could compare standard PONV prophylaxis with or without an NK1R antagonist and evaluate biological co-primary endpoints alongside PONV outcomes, as shown in **Figure 3**.

The fourth implication is patient selection. Breast cancer is biologically attractive because of the SP/TACR1/extracellular RNA/TLR7 evidence. Pancreatic and head-and-neck cancers are attractive because perineural invasion and tumor-neural crosstalk are prominent. Gastrointestinal cancers are operationally attractive because major abdominal surgery and PONV risk are common. Selection should be driven by a measurable SP/NK1R-related biology, not merely by convenience.

## 11 Controversies and knowledge gaps

First, NK1R antagonists have robust anti-vomiting efficacy, but nausea control is less consistent. This pharmacological asymmetry complicates the use of PONV as a single clinical readout and suggests that future trials should separate vomiting, nausea severity, rescue therapy, opioid exposure, and patient-reported recovery.

Second, tumor expression of SP or NK1R is not synonymous with pathway dependence. A tumor may express TACR1 without receiving meaningful neuronal SP input; alternatively, neuronal SP may act on immune or vascular cells rather than tumor cells. Spatial biology is therefore essential.

Third, SP/NK1R signaling may have context-dependent effects. The same pathway can participate in inflammation, pain, immune-cell activation, vascular permeability, tumor-cell stress, and apoptosis-like responses depending on model and dose. A universal “pro-cancer” or “anticancer” label would be misleading.

Fourth, approved antiemetic dosing may not match anticancer pharmacodynamics. A single perioperative dose sufficient to prevent vomiting may be inadequate for sustained tumor-microenvironment modulation. Conversely, repeated dosing for oncologic intent would require evidence not provided by PONV trials or current labeling.

Fifth, immunotherapy resistance studies implicating tumor-associated nerves do not automatically implicate SP. Neural injury can involve adrenergic, cholinergic, sensory neuropeptide, Schwann-cell, cytokine, and myeloid programs. SP/NK1R should be tested within this broader network rather than treated as the dominant neural mediator by default.

Sixth, anesthetic and analgesic strategies can confound interpretation of SP/NK1R biology. Volatile agents, intravenous anesthetics, regional anesthesia, opioids, non-opioid analgesics, and perioperative anti-inflammatory drugs may influence pain, stress responses, immune-cell activation, PONV risk, and postoperative recovery. Future studies should capture these exposures prospectively and analyze them as prespecified covariates rather than treating them as uniform background care (2).

Seventh, countervailing expression and prognostic findings mean that TACR1 or NK1R positivity should not be treated as a universal adverse marker. Null, weak, or inverse associations in specific tumor settings should be incorporated into study design so that perioperative trials enrich for pathway engagement rather than receptor presence alone (23, 24).

## 12 Limitations of current evidence

The current evidence base has several limitations. PONV trials are high-quality clinical studies, but their endpoints are symptoms and short-term recovery rather than tumor biology. Cancer-mechanistic studies provide causal evidence in models, but the perioperative setting introduces anesthetic, analgesic, inflammatory, and timing variables that may not be reproduced in standard tumor models. Human tumor-expression studies are valuable for hypothesis generation but are vulnerable to confounding by stage, treatment, sampling location, and assay variability. The tumor literature also includes countervailing findings in which TACR1/NK1R expression is not clearly adverse, reinforcing the need to distinguish expression from pathway dependence.

Another limitation is compartment ambiguity. SP can be produced by sensory neurons and immune cells, whereas NK1R can be expressed by neural, immune, endothelial, and tumor compartments. Without spatial, cell-type-specific, and temporal measurements, changes in bulk SP or TACR1 expression cannot identify the biologically relevant source-target pair. Finally, the absence of perioperative oncology trials means that recurrence, metastasis, and immunotherapy outcomes remain speculative endpoints for NK1R antagonism.

## 13 Future perspectives

Future studies should progress through a staged translational pathway, as demonstrated in **Table 3**. The first stage should establish perioperative kinetics: how SP, TACR1 expression, NET markers, monocyte activation, extracellular RNA, and TLR signatures change from preoperative baseline to early postoperative recovery. These studies should be tumor-specific and should record pain, opioids, PONV, anesthetic technique, complications, and inflammation.

**Table 3:** Proposed staged translational roadmap.

Stage	Primary goal	Design features	Go/no-go criterion	Tumor-type priority
Stage 1: observational perioperative kinetics	Define temporal changes in SP/NK1R and innate immune markers.	Serial blood sampling, PONV phenotyping, pain/opioid capture, tumor tissue collection.	Reproducible perioperative signal linked to exposure or tumor subtype.	Breast, gastrointestinal, pancreatic, and head-and-neck cancers with measurable perioperative SP/NK1R or innate immune signals.
Stage 2: spatial validation	Identify source-target compartments.	Spatial transcriptomics, multiplex immunofluorescence, nerve and immune-cell mapping.	Co-localization of SP-producing nerves or immune cells with TACR1-positive targets.	Breast and colorectal cancers with TACR1 expression, plus pancreatic and head-and-neck cancers with prominent neural compartments.
Stage 3: causal models	Test whether NK1R manipulation modifies tumor-relevant biology.	Orthotopic surgery models, NK1R antagonist arms, sensory-neuron modulation, immune readouts.	Modification of predefined tumor-neuroimmune biomarkers.	Breast, pancreatic, lung, and colorectal models selected according to pathway activity and surgical feasibility.
Stage 4: biomarker-embedded clinical trial	Test pharmacodynamic effect in humans.	Randomized standard prophylaxis with or without NK1R antagonist; biological co-primary endpoints.	Human biomarker modulation without unacceptable perioperative risk.	Tumor types with evidence of innervation, TACR1 expression, NET activation, or extracellular RNA/TLR signatures.
Stage 5: outcome trial	Evaluate recurrence, metastasis, or immunotherapy-response endpoints.	Tumor-specific multicenter trial with MRD stratification and adjuvant therapy integration.	Predefined clinical benefit with mechanistic concordance.	Tumor-specific multicenter cohorts enriched for MRD detectability and biomarker-defined pathway engagement.

The second stage should use spatial and single-cell technologies to map SP-producing nerves, TACR1-positive tumor cells, innate immune cells, endothelial cells, and extracellular RNA/TLR signatures within resected tumors. This would identify whether the pathway is tumor-intrinsic, immune-mediated, vascular, neural, or mixed in each cancer type.

The third stage should test causality in perioperative models. Orthotopic tumor surgery models should compare NK1R antagonism, sensory-neuron manipulation, opioid exposure, and inflammatory complications, with endpoints including NET formation, myeloid polarization, residual tumor survival, metastatic colonization, and response to immune checkpoint blockade.

The fourth stage should be biomarker-embedded clinical trials. Early trials should prioritize pharmacodynamic endpoints over survival. Later trials can incorporate recurrence-free survival only after demonstrating that perioperative NK1R antagonism modifies a relevant tumor-neuroimmune biomarker in humans.

Finally, the field should develop a standardized perioperative neuroimmune endpoint panel. Such a panel could include PONV phenotypes, pain/opioid metrics, circulating SP, catecholamines, C-reactive protein, interleukin-6, NET markers, monocyte tissue factor, ctDNA, and tumor spatial metrics. Standardization would allow comparison across studies and prevent isolated biomarker claims from being overinterpreted.

## 14 Conclusion

SP/NK1R signaling occupies a narrow but clinically tractable translational position: it is accessible through approved perioperative antiemetics, mechanistically connected to sensory-neuron and innate immune biology, and newly implicated in tumor-neural communication and metastasis. The evidence supports a cautious but actionable hypothesis that SP/NK1R could be evaluated as one candidate perioperative neuro-innate immune checkpoint in selected cancers. The next advance should not be premature claims of anticancer efficacy, but rigorous biomarker-embedded perioperative studies that determine whether NK1R antagonism can modify tumor-relevant neuroimmune biology in humans.
